# Protective and pathogenic dietary factors in bronchitis: Insights from Mendelian randomization

**DOI:** 10.1097/MD.0000000000046632

**Published:** 2025-12-19

**Authors:** Jiawei Wang, Zekun Du, Zhangyu Lu, Ting Yang

**Affiliations:** a West China School of Medicine, Sichuan University, Chengdu, China; b Department of Pulmonary and Critical Care Medicine, West China Hospital of Sichuan University, Chengdu, China.

**Keywords:** bronchitis, diet habits, Mendelian randomization

## Abstract

Previous studies have suggested a potential association between dietary habits and bronchitis but the exact nature of this relationship remains unclear. This study aims to elucidate the causal relationship between these 2 factors using a Mendelian randomization (MR) approach. We performed a two-sample MR analysis using genome-wide association studies data from the UK Biobank for 231 dietary phenotypes and FinnGen for bronchitis cases. Single nucleotide polymorphisms were selected as IVs after rigorous filtering, including significance thresholds (*P* < 5e‐6), *F*-statistics (>10), and tests for pleiotropy and reverse causality. The inverse variance weighted method was the primary analytical approach, with MR-Egger, weighted median, and other methods for sensitivity checks. Our MR analysis revealed significant causal effects of dietary phenotypes on bronchitis. Dried fruit intake (odds ratio [OR] = 0.963, 95% confidence intervals [CI]: 0.940–0.986, *P* = .002), beef intake (OR = 0.954, 95% CI: 0.919–0.991, *P* = .015), and fresh fruit intake (OR = 0.977, 95% CI: 0.945–0.999, *P* = .043) exhibited protective effects against bronchitis. Conversely, alcohol intake compared to 10 years prior increased risk (OR = 1.055, 95% CI: 1.001–1.111, *P* = .042). Sensitivity analyses confirmed robustness. This study utilized publicly available genome-wide association studies data to explore the causal relationships between dietary habits and bronchitis using MR methods. It found dried and fresh fruit, and beef intake protect against bronchitis, while alcohol increases risk. These findings offer insights for future research and potential dietary interventions for bronchitis management.

## 1. Introduction

Bronchitis is a common respiratory disease characterized by acute or chronic nonspecific inflammation of the bronchial mucosa and surrounding tissues that typically manifests as cough and sputum production. It can be classified as an acute, chronic, or plastic bronchitis. Acute bronchitis is primarily caused by viral infections,^[1]^ while chronic bronchitis, a major pathological feature of chronic obstructive pulmonary disease, is associated with prolonged exposure to irritants such as tobacco smoke and environmental pollutants.^[2]^ Although rare, plastic bronchitis is a potentially fatal form of bronchitis.^[3]^ Chronic bronchitis affects approximately 10.1% of individuals aged ≥40 years globally, and chronic obstructive pulmonary disease was the third leading cause of death worldwide in 2019, representing a significant healthcare burden, especially in low- and middle-income countries.^[2]^ Complications, such as respiratory failure and increased susceptibility to lung infections, underscore the serious public health impact of bronchitis. In the United States alone, acute bronchitis accounts for approximately 100 million medical visits annually, and the widespread use of antibiotics for its treatment has contributed to the growing issue of antibiotic resistance, leading to over 2 million antibiotic-resistant infections and 23,000 deaths annually.^[4]^ Given the public health significance of bronchitis, further research is critical for the development of effective prevention and treatment strategies.

In recent years, increasing attention has been directed toward the relationship between dietary habits and diseases.^[5]^ For instance, Wen et al identified that adherence to the dietary approaches to stop hypertension diet, rich in antioxidants and anti-inflammatory components such as flavonoids, vitamin A, and vitamin E, could alleviate the inflammatory symptoms of chronic bronchitis.^[6]^ Similarly, Wu et al demonstrated that flavonoid-rich diets reduce symptoms of chronic respiratory diseases, possibly by inhibiting pro-inflammatory enzymes and cytokines, reducing reactive oxygen species (ROS) production, and interrupting inflammation-related signaling pathways.^[7]^ In an animal study, a diet high in docosahexaenoic acid led to significantly increased levels of docosahexaenoic acid-derived resolvins in bronchoalveolar lavage fluid and reduced levels of tumor necrosis factor alpha in mice with induced lung inflammation, as well as lower plasma levels of inflammatory mediators, compared with a normal diet.^[8]^ Although such studies suggest that dietary patterns may be linked to bronchitis and propose potential molecular mechanisms, limitations, such as incomplete dietary surveys and insufficient sample sizes, hinder the strength of these findings. Thus, more comprehensive research is required to clarify the relationship between dietary habits and bronchitis.

Mendelian randomization (MR) offers a robust approach for exploring causal relationships between exposures and outcomes based on Mendel law of independent assortment.^[9]^ Using genetic variations as instrumental variables (IVs), MR can provide reliable evidence of causality between exposures and outcomes. Single nucleotide polymorphisms (SNPs) associated with exposure were selected as IVs to estimate the causal effect of genetically proxied exposure on outcomes. This method can generate results analogous to those of randomized controlled trials because the random allocation of genetic variants during meiosis minimizes confounding factors and reduces the risk of reverse causality.^[10]^ Moreover, MR addresses the ethical and economic challenges associated with randomized controlled trials.^[11]^ This study aimed to use MR, employing appropriate IVs, to investigate the relationship between dietary habits and bronchitis, providing novel insights and evidence-based guidance for future research.

## 2. Methods

### 2.1. Study design

**Figure 1.**
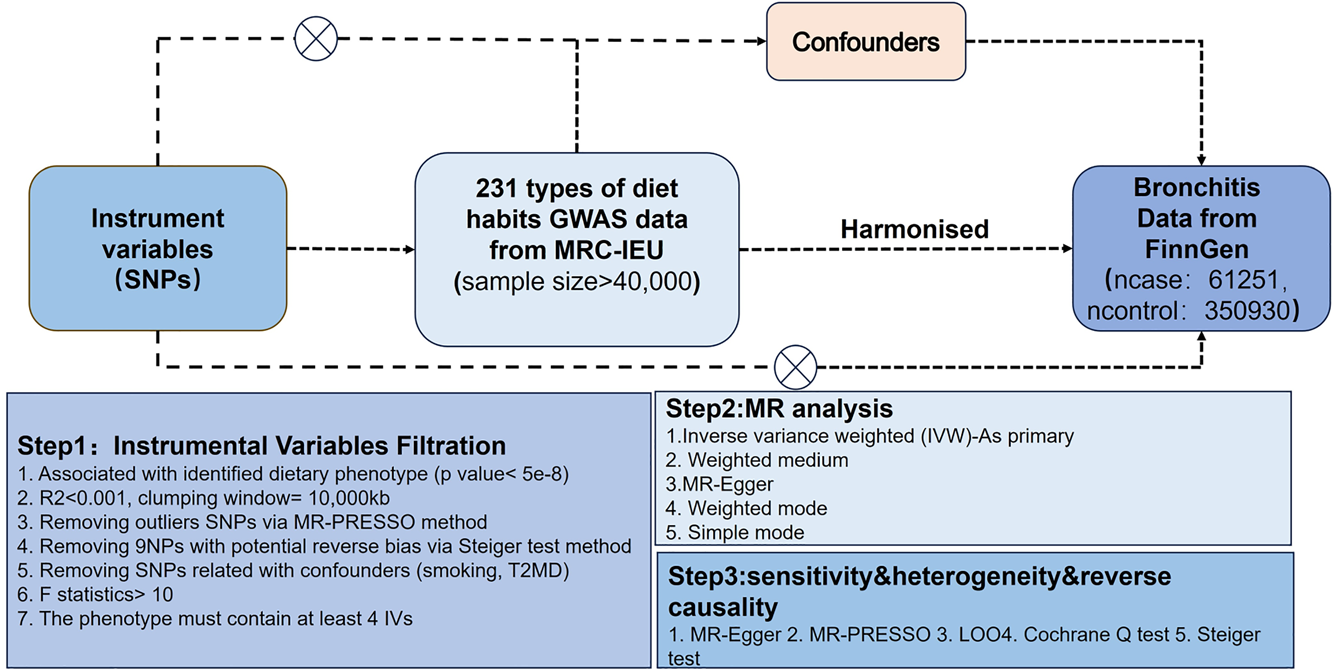
Overall study design. The arrow indicates the direction of action and influence. GWAS = genome-wide association studies; LOO = leave-one-out analysis; SNP = single-nucleotide polymorphism; T2DM = type 2 diabetes mellitus.

This study utilized publicly available genome-wide association study (GWAS) data on dietary habits and bronchitis. A total of 231 dietary habits, sourced from the UK Biobank, were considered exposure variables, while bronchitis was the outcome variable, with data derived from FinnGen. This study employed a two-sample MR approach to comprehensively explore the causal relationship between dietary habits and bronchitis. First, we extracted SNPs strongly associated with dietary habits as IVs. These 231 dietary habits were used as exposure data, while bronchitis served as outcome data, and we assessed the causal effects of dietary habits on bronchitis. In conducting the MR analysis, we strictly adhered to 3 core assumptions: the IVs must be strongly associated with the exposure, the IVs must not be associated with any confounders, and the IVs must affect the outcome only through the exposure and not directly. A rigorous selection process was applied to extract IVs, and 5 MR analysis methods with quality control measures were employed to ensure the robustness of the results. Furthermore, the study followed the STROBE-MR guidelines for reporting findings.^[12]^ The overall study process is illustrated in Figure 1.

### 2.2. Data sources

#### 2.2.1. GWAS data for dietary habits

To investigate the causal relationship between dietary habits and bronchitis, we used GWAS data from 231 dietary phenotypes, obtained from the UK Biobank (https://gwas.mrcieu.ac.uk/). The UK Biobank is a prospective cohort study that has collected deep genetic and phenotypic data from approximately 500,000 participants, aged 40 to 69 years, from the UK. Participants provided a range of biological samples, including blood, urine, and saliva, which were used for genotyping and other biomarker assessments.^[13]^ Dietary habits were recorded using questionnaires, and GWAS data on dietary habits were obtained using custom genotyping arrays, high-quality data integration, and advanced phasing and genotype imputation methods (such as SHAPEIT3 and the HRC reference panel). Statistical models, including linear mixed models, were employed to perform GWAS, resulting in dietary habit data covering 231 phenotypes, including Beef intake, Poultry intake, Bacon intake, and others.

#### 2.2.2. GWAS data for bronchitis

The GWAS data for bronchitis used in this study were sourced from FinnGen, a public–private research project that integrates genotypic data from Finnish biobanks with digital health records from Finland’s health registries (https://r10.finngen.fi/pheno/BRONCHITIS). The dataset comprises genotypic information from approximately 500,000 individuals in Finland, coupled with national health registry data.^[14]^ The bronchitis-related dataset included 61,251 cases and 350,930 controls.

### 2.3. IV SNPs filtration

To meet the assumptions of the MR, a stringent process for extracting IVs was employed. First, SNPs associated with smoking and type II diabetes were excluded from the GWAS catalog. To ensure strong correlations between the SNPs and exposure, a significance threshold of *P* < 5e‐6 was applied, and *F*-statistics were calculated, including only data with *F* > 10 in the analysis.^[15]^ To control for linkage disequilibrium, we set a window size of 10,000 kb and excluded SNPs with an *R*² > 0.001. Further checks for pleiotropy and reverse causality were performed using the MR-PRESSO analysis and Steiger test, respectively,^[16,17]^ and only SNPs with MR-PRESSO *P*-values >.05 and Steiger *P*-values <.05. Additionally, minor allele frequency (MAF) was considered, and only SNPs with MAF > 0.01 were included to minimize the risks associated with ambiguous and palindromic sequences.^[18]^ Finally, only phenotypes with >4 valid IVs were retained for the analysis.

### 2.4. MR analysis

Five MR methods were applied to assess the causal relationship between dietary habits and bronchitis: inverse variance weighted (IVW),^[19]^ MR-Egger regression,^[19]^ weighted median,^[10]^ simple mode, and weighted mode. These methods used SNPs as IVs in two-sample MR analysis. Because the IVW method is considered the most robust and provides moderately accurate estimates in the presence of heterogeneity, it was selected as the primary MR analysis method, with the other 4 methods used as supplementary analyses.^[15]^ If the *P*-value for IVW was <.05, and the odds ratio (OR) directions were consistent across the other methods, the results were considered robust.

### 2.5. Sensitivity, heterogeneity, and reverse causality

We conducted sensitivity analyses using MR-PRESSO, Egger intercept, and leave-one-out (LOO) methods. MR-PRESSO was used to assess global pleiotropy through repeated sampling,^[16]^ while the Egger intercept was used to test for horizontal pleiotropy by examining whether the intercept was significantly different from zero.^[17]^ The LOO method was used to assess the influence of individual SNPs on the outcome by iteratively removing 1 SNP at a time and visualizing the results in a forest plot. If both the MR-PRESSO and Egger intercept *P*-values were >.05 and no outliers were identified in the LOO analysis, the risk of pleiotropy was considered low.

For heterogeneity analysis, we applied Cochran *Q* test for both the IVW and MR-Egger methods.^[18]^ A *P*-value >.05 for both methods indicated a low risk of heterogeneity. To assess the risk of reverse causality, we performed the Steiger test. If the Steiger test *P*-value was <.05, the risk of reverse causality was considered low, ensuring the robustness of our findings.

### 2.6. Data processing

All statistical analyses were conducted using R software (version 4.3.1; R Foundation for Statistical Computing, Vienna, Austria) (http://www.r-project.org). MR analysis, sensitivity analysis, and data visualization were performed using the “TwoSampleMR” (version 0.5.6) and “MR-PRESSO” packages.^[20]^ Additional statistical analyses and visualizations were carried out using the “MendelianRandomization” and “ggplot2” R packages. The magnitude of causal effects was expressed as OR with 95% confidence intervals (CI).

## 3. Results

### 3.1. IVs filtration

Following the rigorous IV extraction process outlined in the methods section, we ultimately identified 30 dietary phenotypes from the 231 dietary habits GWAS data from the UK Biobank diet-related questionnaire survey, including dried fruit intake, beef intake, alcohol intake versus 10 years previously, fresh fruit intake, bread intake, snackpot intake, herbal tea intake, Scotch egg intake, lamb/mutton intake, cheese intake, water intake, unsalted peanuts intake, cereal intake, average weekly spirits intake, non-oily fish intake, salad/raw vegetable intake, oily fish intake, green tea intake, average weekly beer plus cider intake, Sushi intake, coffee intake, average weekly red wine intake, Tofu intake, Pork intake, alcohol intake frequency, tea intake, processed meat intake, pancake intake, poultry intake, and cooked vegetable intake, and included 696 SNPs in the analysis. These SNPs met the criteria of MAF >0.01, thereby excluding the ambiguous and palindromic SNPs. All selected SNPs also satisfied the threshold of *R*² > 0.001 and *F*-statistics >10, ensuring the exclusion of weak instruments. Additionally, MR-PRESSO and Steiger tests confirmed the absence of horizontal pleiotropy and reverse causality, respectively. Therefore, the selected SNPs were robust, independent, and suitable for MR analysis. The complete list of SNPs used in MR analysis is shown in Table S1, Supplemental Digital Content, https://links.lww.com/MD/Q952.

### 3.2. The results of dietary habits and bronchitis

MR analysis, conducted using 5 different methods and supported by sensitivity, heterogeneity, and reverse causality tests, revealed that 4 dietary phenotypes demonstrated significant causal effects on bronchitis. Based on the results of the IVW method, dried fruit intake (*P* = .002, OR = 0.963, 95% CI = 0.940–0.986), beef intake (*P* = .015, OR = 0.954, 95% CI = 0.919–0.991), and fresh fruit intake (*P* = .043, OR = 0.977, 95% CI = 0.945–0.999) exhibited protective effects against bronchitis, whereas Alcohol intake versus 10 years previously (*P* = .042, OR = 1.055, 95% CI = 1.001–1.111) was associated with an increased risk of bronchitis.

**Figure 2.**
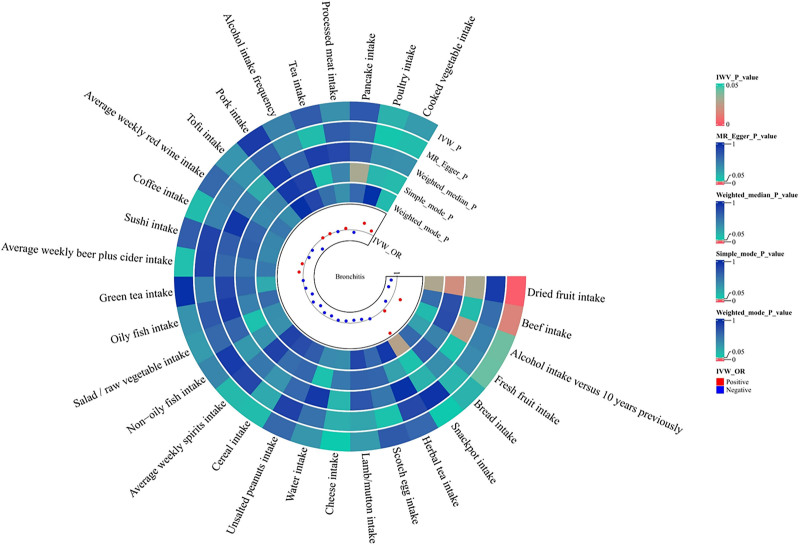
Using 5 Mendelian randomization methods, the causal effects of all dietary habits included in the analysis were estimated. Blue dots represent the directionality of the OR values in the IVW method as protective, while red dots represent the directionality of the OR values in the IVW method as aggravating. The circles from the outside in represent the *P*-values of IVW, MR Egger, weighted median, simple mode, and weighted mode. The outermost digit of each circle represents the ID of each dietary habit. IVW = inverse variance weighting, MR = Mendelian randomization, OR = odds ratio.

**Figure 3.**
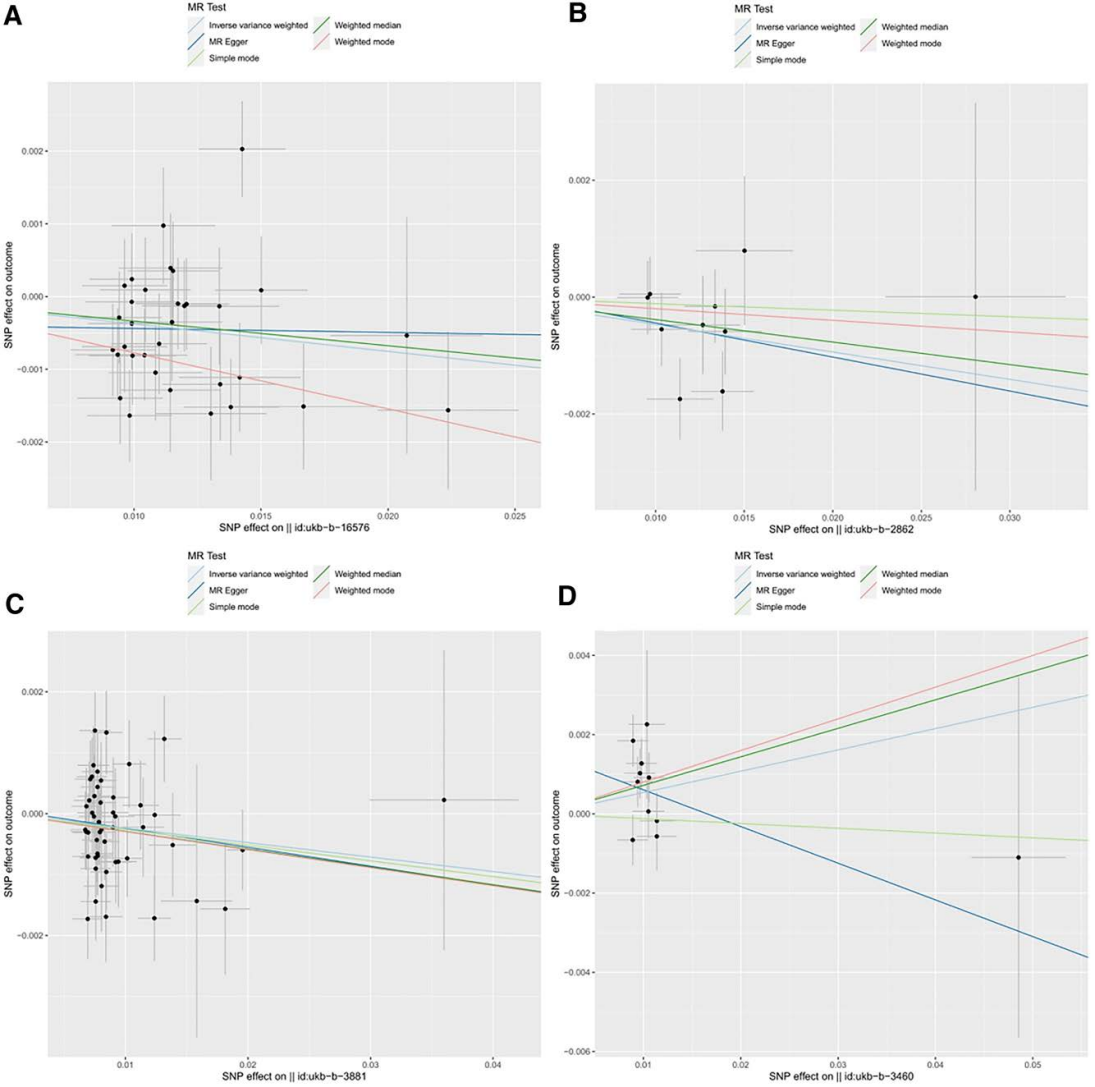
Scatter plots of positive results under the 4 IVW methods. (A) Dried fruit intake; (B) beef intake; (C) fresh fruit intake; (D) alcohol intake versus 10 years previously. IVW = inverse variance weighting, SNP = single-nucleotide polymorphism.

For dried fruit intake, beef intake, and fresh fruit intake, the ORs from all 5 MR methods were directionally consistent, confirming the robustness of the protective effects. However, analysis of alcohol intake versus 10 years previously yielded more complex results. While the IVW, weighted median, and weighted mode methods indicated a positive association, neither the MR-Egger (*P* = .496, OR = 0.912, 95% CI = 0.706–1.178) nor simple mode (*P* = .830, OR = 0.988, 95% CI = 0.087–1.100) methods produce statistically significant results, suggesting that caution is warranted when interpreting the relationship between alcohol intake and bronchitis. The detailed results can be found in Figures 2 and 3, and additional analytical details are provided in Tables S2 and S3, Supplemental Digital Content, https://links.lww.com/MD/Q952.

### 3.3. Sensitivity, heterogeneity, and reverse causality

**Figure 4.**
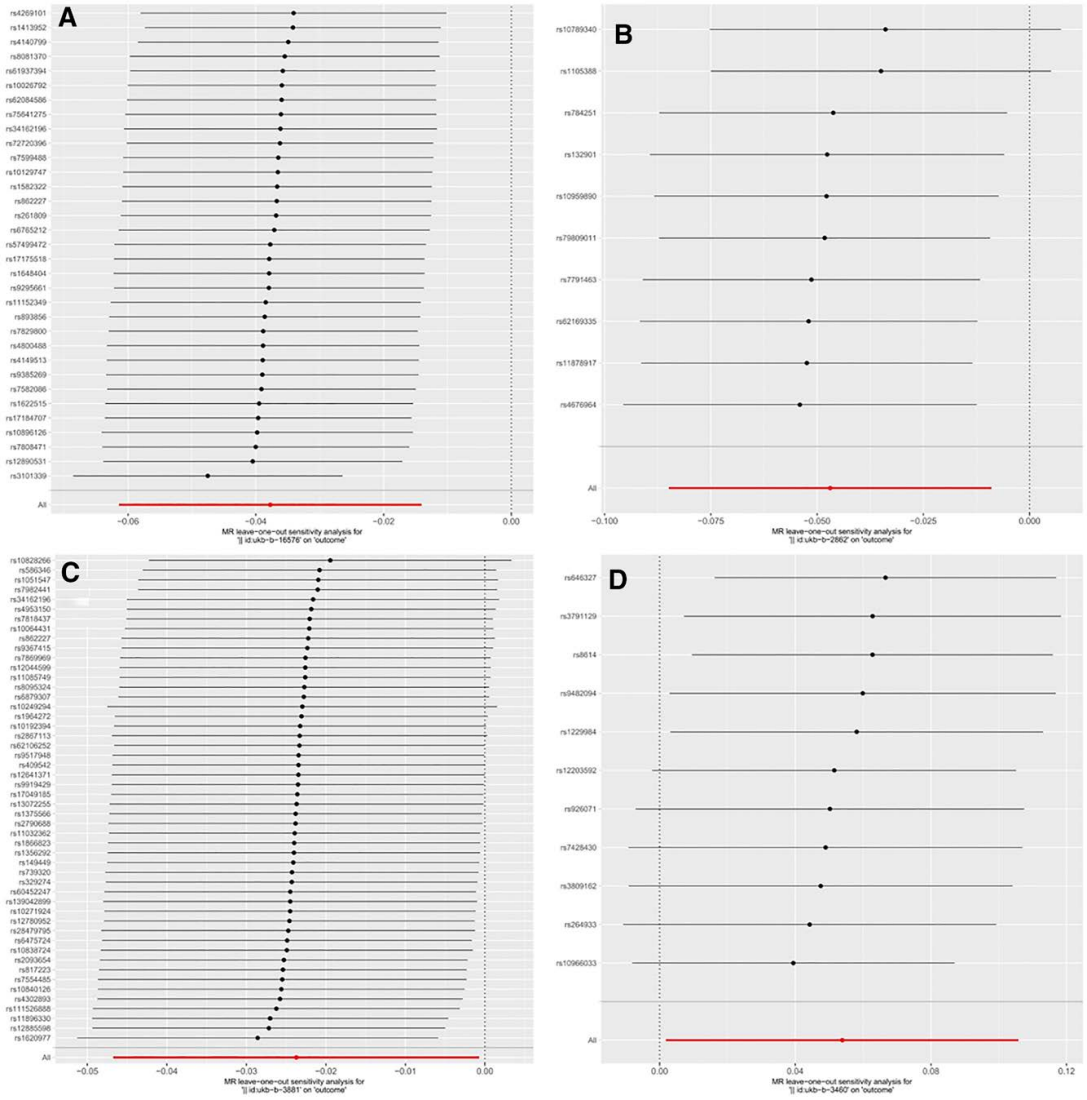
Forest plots of leave-one-out analysis of positive results under the 4 IVW methods. (A) Dried fruit intake; (B) beef intake; (C) fresh fruit intake; (D) alcohol intake versus 10 years previously. IVW = inverse variance weighting.

In the sensitivity analysis, the MR-Egger intercept and MR-PRESSO global tests indicated *P*-values >.05, for dried fruit intake, beef intake, fresh fruit intake, and alcohol intake versus 10 years previously, suggesting no evidence of horizontal pleiotropy. Similarly, the LOO analysis did not identify any outlier SNPs, further indicating low sensitivity risk. Forest plots from the LOO analysis are provided in Figure 4, and the results of the MR-Egger intercept and MR-PRESSO global tests are detailed in Table S2, Supplemental Digital Content, https://links.lww.com/MD/Q952.

For heterogeneity analysis, Cochran *Q* test conducted through both the IVW and MR-Egger methods showed *P*-values > .05 for dried fruit intake, beef intake, fresh fruit intake, and alcohol intake versus 10 years previously, indicating a low risk of heterogeneity and good homogeneity in the results.

Lastly, the reverse causality test using the Steiger test revealed *P*-values < .05 for all 4 phenotypes, confirming that the risk of reverse causality was low and that the directionality of the effects was consistent with the exposure–outcome relationships examined in this study.

## 4. Discussion

Bronchitis, characterized by inflammation of the bronchial mucosa, is a common respiratory condition. Acute bronchitis is primarily caused by viral infections, but treatment often relies on antibiotics, contributing to the serious issue of antibiotic overuse.^[21]^ Chronic bronchitis, on the other hand, is progressive and difficult to manage,^[22]^ with an estimated 3.4% to 22% of adults affected. In the United States, approximately 10 million individuals suffer from chronic bronchitis,^[23]^ emphasizing the significant impact of this disease on public health. There is growing evidence that dietary habits may be linked to bronchitis, with some studies suggesting that components of the Mediterranean diet, known for their anti-inflammatory and antioxidant properties, can alleviate the symptoms of chronic bronchitis through epigenetic modifications.^[24]^ Similarly, flavonoids, coumarins, and phenolic compounds found in various foods have been shown to exhibit anti-inflammatory effects on bronchial inflammation.^[25]^ However, previous studies have often focused on specific foods or dietary patterns and lack comprehensive evidence of a causal relationship between dietary habits and bronchitis. To address these limitations, this study utilized publicly available GWAS data and applied 5 MR methods to explore the causal effects of various dietary habits on bronchitis. Ultimately, 4 dietary habits were identified to have significant associations with bronchitis, with dried fruit intake, beef intake, and fresh fruit intake exhibiting protective effects, while alcohol intake versus 10 years previously was associated with an increased risk of bronchitis.

Dried fruit contains several components with potential anti-inflammatory properties, including unsaturated fatty acids, vitamins A and E, and polyphenolic compounds.^[26]^ Notably, vitamin E comprises 8 fat-soluble compounds, including α-, β-, γ-, and δ-tocopherols and tocotrienols.^[27]^ The antioxidant properties of vitamin E stem from its ability to form unstable tocopherol radicals during reactions with ROS and/or RNS, which subsequently undergo radical–radical coupling, thereby neutralizing oxidative stress.^[28]^ Tocotrienols, especially δ- and γ-tocotrienols, have been shown to have potent anti-inflammatory effects by regulating key inflammatory signaling pathways, such as NF-κB and the NLRP3 inflammasome, while also reducing oxidative stress and ROS production.^[29]^ Polyphenols, another class of compounds found in dried fruits, have also been shown to attenuate inflammation.^[30]^ Wu et al reported that high polyphenol intake could reduce inflammation through the epigenetic regulation of DNA methylation enzymes, thereby modulating DNA methylation levels.^[31]^ This in turn protects cells and biomolecules from oxidative stress and inflammation.^[32]^ Although previous studies have suggested that dried fruit intake may benefit respiratory diseases,^[33]^ the limited scope of dietary exposure has left the relationship between dietary habits and bronchitis underexplored. Using European population data and MR analysis, this study provides robust evidence supporting the protective effect of Dried fruit intake (*P* = .002, OR = 0.963, 95% CI = 0.940–0.986) against bronchitis, consistent with earlier research and offering valuable dietary guidance for bronchitis prevention.

Additionally, fresh fruit intake (*P* = .043, OR = 0.977, 95% CI = 0.945–0.999) had a protective effect against bronchitis. Fresh fruits are rich in dietary fiber, potassium, vitamin C, flavonoids, and terpenes, all of which play important roles in promoting respiratory health.^[34]^ Wang et al found that increased fruit consumption could lower mortality related to respiratory diseases,^[35]^ and Sdona et al demonstrated that increasing fruit intake from childhood improved respiratory function later in life.^[36]^ The protective effect of fruits may be attributed to their high content of anti-inflammatory and antioxidant compounds.^[37]^ For example, figs, are rich in polyphenolic compounds, which, as previously discussed, have anti-inflammatory properties.^[38]^ Liu et al found that fig polyphenol extracts inhibited nitric oxide production, thereby reducing reactive nitrogen species and exerting anti-inflammatory effects.^[39]^ Citrus fruits are abundant in vitamin C, which plays a crucial role in reducing oxidative stress and inflammation by inhibiting NF-κB activation and lowering the production of pro-inflammatory cytokines such as tumor necrosis factor alpha and IFN-γ while increasing anti-inflammatory cytokines like IL-10.^[40–42]^ Pineapple, another fruit rich in bromelain, has been reported to aid in treating bronchitis.^[43]^ Although direct studies on the link between fruit intake and bronchitis are limited, MR analysis in this study confirmed the protective effect of fresh fruit intake, supporting the hypothesis that fruit consumption may delay the progression of bronchitis, although further research is needed to explore the underlying mechanisms.

Beef intake (*P* = .015, OR = 0.954, 95% CI = 0.919–0.99) also showed potential preventive effects against bronchitis. Beef is a common dietary staple, and in 2022, the U.S. exported $11.7 billion worth of beef and beef products.^[44]^ It contains taurine, creatine, carnosine, 4-hydroxyproline, and various proteins, many of which have demonstrated anti-inflammatory effects.^[45]^ Both carnosine and 4-hydroxyproline have been found to mitigate inflammation.^[46,47]^ Additionally, Wood et al suggested that beef intake can elevate plasma glutamine concentrations and reduce C-reactive protein levels, thus reducing inflammation.^[48]^ Although previous studies have not directly linked beef consumption to bronchitis, some studies suggested that diets low in red meat intake could offer protection against respiratory diseases.^[49]^ The findings of this study highlight the need for further investigation of the relationship between beef intake and bronchitis.

Conversely, alcohol consumption has been associated with an increased risk of several diseases, including colorectal cancer, cardiovascular diseases, and osteoarthritis.^[50–52]^ This study found that alcohol intake increased the risk of bronchitis (*P* = .042, OR = 1.055, 95% CI = 1.001–1.111). Research by Lovelock et al revealed that alcohol and its metabolite acetaldehyde could activate Toll-like receptors (TLR 2, 3, and 4) and NLRP3 inflammasomes in immune cells, triggering systemic inflammatory responses.^[53]^ Additionally, alcohol can promote bacterial overgrowth in the gastrointestinal tract, leading to the production of lipopolysaccharides, which activate macrophages and other innate immune cells, exacerbating inflammation.^[54]^ A study conducted in Denmark also reported a significant association between alcohol consumption and bronchitis (*P* < .01),^[55]^ consistent with the results of this study. However, as the MR-Egger (*P* = .496, OR = 0.912, 95% CI = 0.706–1.178) and simple mode (*P* = .830, OR = 0.988, 95% CI = 0.087–1.100) methods did not provide statistically significant results, further research is needed to clarify the relationship between alcohol intake and bronchitis.

This study has several strengths. First, it leveraged large-scale European population data, to enhance the representativeness and credibility of the findings. Second, the use of genetic variants (SNPs) as IVs reduces the likelihood of bias due to reverse causality and confounding factors.^[10]^ Third, multiple quality control methods, including MR-PRESSO, Egger intercept, and Cochran *Q* test, ensured that heterogeneity and pleiotropy were adequately addressed, resulting in robust findings. Thus, this study provides valuable insights into the causal relationships between 231 dietary habits and bronchitis, overcoming limitations in previous studies related to small sample sizes and confounding factors, and offers strong evidence for future research.

However, this study has certain limitations. First, although the sample size is large, the data were derived primarily from European populations, limiting the generalizability of the findings to non-European populations.^[56]^ There is also the potential for population stratification. Second, stringent filtering criteria may have excluded some important phenotypes, preventing a more comprehensive exploration of the relationship between dietary habits and bronchitis. Moreover, this study did not account for potential confounders such as gender and age. Future research should aim to include non-European populations, reanalyze omitted phenotypes, and stratify data by demographics such as gender and age to ensure more rigorous conclusions. High-quality MR studies are required to further investigate these relationships.

## 5. Conclusion

This study utilized publicly available GWAS data to explore the causal relationships between various dietary habits and bronchitis using MR methods. The findings revealed that intake of dried fruit, fresh fruit, and beef have protective effects against bronchitis, while alcohol intake versus 10 years previously increases the risk of bronchitis. These results provide valuable evidence for future research in this field and offer dietary recommendations that may aid in the prevention and management of bronchitis. The identification of these dietary habits as potential modifiers of bronchitis progression highlights the need for further investigation of their mechanistic roles in respiratory health. Moreover, this study underscores the importance of comprehensive dietary patterns in the context of bronchitis, offering new directions for dietary interventions aimed at mitigating bronchitis symptoms and progression.

## Acknowledgments

We express our sincere gratitude to the IEU Open GWAS Project for their invaluable efforts in collecting and archiving the GWAS summary statistics related to dietary intake. We also extend our appreciation to the FinnGen Consortium for generously providing us with the raw data about bronchitis.

## Author contributions

**Conceptualization:** Jiawei Wang, Zekun Du, Zhangyu Lu.

**Data curation:** Jiawei Wang, Zekun Du, Zhangyu Lu.

**Formal analysis:** Jiawei Wang, Zekun Du, Zhangyu Lu.

**Investigation:** Jiawei Wang, Zekun Du, Zhangyu Lu.

**Methodology:** Jiawei Wang, Zekun Du, Zhangyu Lu.

**Project administration:** Zekun Du, Zhangyu Lu, Ting Yang.

**Resources:** Jiawei Wang, Zekun Du, Zhangyu Lu.

**Software:** Jiawei Wang, Zekun Du, Zhangyu Lu.

**Supervision:** Ting Yang.

**Validation:** Jiawei Wang, Zekun Du, Zhangyu Lu.

**Visualization:** Jiawei Wang, Zekun Du, Zhangyu Lu.

**Writing – original draft:** Jiawei Wang, Zekun Du, Zhangyu Lu, Ting Yang.

**Writing – review & editing:** Jiawei Wang, Zekun Du, Zhangyu Lu, Ting Yang.

## Supplementary Material


